# Trends in the antimicrobial susceptibility among Chinese neonates from 2012 to 2021: a multicenter study

**DOI:** 10.1186/s13756-024-01440-2

**Published:** 2024-07-30

**Authors:** Zhanghua Yin, Jintong Tan, Huafei Huang, Jianyuan Zhao, Xiaohui Gong, Jing Li, Chao Chen, Fei Luo, Xiaoyi Huang, Huaiyan Wang, Hongyan Lu, Mingfu Wu, Renqiang Yu, Xiaoping Lei, Qian Zhang, Fengdan Xu, Ning Li, Hong Jiang, Jianhua Fu, Rui Cheng, Yan Chen, Yongjun Zhang

**Affiliations:** 1 Department of Pediatrics, Xinhua Hospital, School of Medicine, Shanghai Jiao Tong University, 1665 Kongjiang Road, Yangpu District, Shanghai, 200092 China; 2 Department of Neonatology, Jiaxing University Affiliated Women and Children Hospital, 2468 Zhonghuan East Road, Nanhu District, Jiaxing, 314000 China; 3 Institute for Developmental and Regenerative Cardiovascular Medicine, MOE-Shanghai Key Laboratory of Children’s Environmental Health, Xinhua Hospital, School of Medicine, Shanghai Jiao Tong University, Shanghai, China; 4 Department of Neonatology, School of Medicine, Children’s Hospital of Shanghai, Shanghai Jiao Tong University, Shanghai, China; 5 Department of Neonatology, Shanghai Children’s Medical Center, Shanghai Jiao Tong University School of Medicine, Shanghai, China; 6 Department of Neonatology, Shanghai first maternity and infant hospital, Shanghai, China; 7 Department of Neonatology, Children’s Hospital of Fudan University, Shanghai, China; 8 Department of Neonatology, Obstetrics & Gynecology Hospital of Fudan University, Shanghai, China; 9 Department of Neonatology, School of Medicine, International Peace Maternity and Child Health Hospital, Shanghai Jiao Tong University, Shanghai, China; 10 Department of Neonatology, Changzhou Medical Center, Changzhou Maternal and Child Health Care Hospital, Nanjing Medical University, Changzhou, China; 11 Department of Neonatology, Affiliated Hospital of Jiangsu University, Zhenjiang, China; 12 Department of Neonatology, Affiliated Hospital of Yangzhou University, Yangzhou, China; 13 Department of Neonatology, Affiliated Women’s Hospital of Jiangnan University, Wuxi, China; 14 Department of Neonatology, The Affiliated Hospital of Southwest Medical University, Luzhou, China; 15 Department of Neonatology, The First Affiliated Hospital of Zhengzhou University, Zhengzhou, China; 16 Department of Neonatology, Dongguan Children’s Hospital Affiliated to Guangdong Medical University, Dongguan, China; 17 Department of Neonatology, The Affiliated Hospital of Qingdao University, Qingdao, China; 18 Department of Neonatology, Shengjing Hospital of China Medical University, Shenyang, China; 19 Department of Neonatology, Children’s Hospital of Nanjing Medical University, 72 Guangzhou Road, Gulou District, Nanjing, 210008 China; 20 Shanghai Institute for Pediatric Research, Xinhua Hospital, Shanghai Jiao Tong University School of Medicine, 1665 Kongjiang Road, Yangpu District, Shanghai, 200092 China

**Keywords:** Full-term neonates, Invasive bacterial infections, Antimicrobial susceptibility, Minimum inhibitory concentration, Trends, China

## Abstract

**Background:**

Antibiotic resistance is a serious global public health issue. However, there are few reports on trends in antimicrobial susceptibility in Chinese neonates, and most of the existing evidence has been derived from adult studies. We aimed to assess the trends in antimicrobial susceptibility of common pathogens in full-term neonates with invasive bacterial infections (IBIs) in China.

**Methods:**

This cross-sectional survey study analyzed the antimicrobial susceptibility in Chinese neonates with IBIs from 17 hospitals, spanning from January 2012 to December 2021. Joinpoint regression model was applied to illustrate the trends and calculate the average annual percentage change (AAPC). Using Mantel-Haenszel linear-by-linear association chi-square test, we further compared the antibiotic minimum inhibitory concentrations (MICs) by pathogens between 2019 and 2021 to provide precise estimates of changes.

**Results:**

The proportion of Escherichia coli with extended-spectrum-beta-lactamase-negative strains increased from 0.0 to 88.5% (AAPC = 62.4%, 95% confidence interval (CI): 44.3%, 82.9%), with two breakpoints in 2014 and 2018 (p-trend < 0.001). The susceptibility of group B Streptococcus (GBS) to erythromycin and clindamycin increased by 66.7% and 42.8%, respectively (AAPC = 55.2%, 95% CI: 23.2%, 95.5%, p-trend = 0.002; AAPC = 54.8%, 95% CI: 9.6%, 118.6%, p-trend < 0.001), as did Staphylococcus aureus to penicillin (AAPC = 56.2%; 95% CI: 34.8%, 81.0%, p-trend < 0.001). However, the susceptibility of Enterococcus spp. to ampicillin declined from 100.0 to 25.0% (AAPC = − 11.7%, 95% CI: − 15.2%, − 8.1%, p-trend < 0.001), and no significant improvement was observed in the antibiotic susceptibility of Escherichia coli to ampicillin, gentamicin, and cephalosporin. Additionally, the proportion of GBS/Staphylococcus aureus with relatively low MIC values for relevant antibiotics also increased in 2021 compared to 2019.

**Conclusions:**

Antimicrobial susceptibility of the most prevalent pathogens in full-term neonates seemed to have improved or remained stable over the last decade in China, implying the effectiveness of policies and practice of antibiotic stewardship had gradually emerged.

**Supplementary Information:**

The online version contains supplementary material available at 10.1186/s13756-024-01440-2.

## Background

Antimicrobial resistance poses a pressing threat to human health, affecting all regions at all income levels [1, 2]. Low- and middle-income regions are most impacted [1]. From 1998 to 2016, the proportion of the most prevalent gram-negative pathogens with extended-spectrum beta-lactamase (ESBL) or fluoroquinolone resistance had expanded rapidly, and methicillin-resistant Staphylococcus aureus (MRSA) had emerged within Sub-Saharan Africa [3]. According to a study by China’s National Center for Antimicrobial Resistance, some pathogenic strains, such as Escherichia coli, had a high proportion (60–80%) of resistance to ciprofloxacin over a 7-year period starting in 1994, which is still increasing at an average annual growth rate of approximately 15% [4]. The UN General Assembly’s High-Level Meeting gradually moved the focus onto antimicrobial resistance, and in 2016 provided guidance to political leaders on how to promote sustainable action on antimicrobial resistance. This guidance included the appropriate use of antibiotics in human and animal health, eradication of untreated effluent, prevention and control of infections, and supply of quality-assured and affordable antimicrobials [5]. In response, the Chinese government instituted a series of critical policies to mitigate the escalating trend of antibiotic resistance, including strengthening the supervision of antibiotic marketing paradigm, vigorously promoting pediatric vaccination, as well as supporting the development of new antimicrobial agents [6].

Existing reports of antibiotic resistance include single pathogen species or single-center designs and the information released by the China Antimicrobial Surveillance Network (https://www.carss.cn/Report) are annual cross-sectional surveys [7, 8]. These reports do provide some support regarding trends in antimicrobial resistance; however, they lacked long-term tracking and comprehensive assessments of this issue. Moreover, the majority of these studies focused on adults or adolescents, with limited specific research regarding antibiotic resistance in neonatal cohorts. Compared to adults, neonatal bacterial infections exhibit unique characteristics in terms of bacterial spectrum and antibiotic selection [9, 10]. As the immune function of newborns is still developing, they are more susceptible to a broad range of pathogens, rendering them more vulnerable to invasive bacterial infections (IBIs) and necessitating more frequent administration of antibiotics [11]. Furthermore, IBIs during the neonatal period are confirmed to be associated with high rates of mortality and sequelae, which accounted for 12.8% of global newborn deaths in 2010, thus necessitating heightened attention from clinicians [12, 13]. Having a clear understanding of the antibiotic resistance profile will have direct implications for antibiotic selection, supervision, and disease burden reduction. Considering the existing evidence of increasing antimicrobial resistance, we urgently attempted to explore the temporal trajectory of antibiotic susceptibility in Chinese newborns between 2012 − 2021, intending to optimize empirical anti-infection strategies, assess the effectiveness of policies, and facilitate subsequent revisions.

## Methods

### Study design

This was a cross-sectional multicenter survey that included full-term neonates with IBIs in 17 tertiary hospitals located in eight municipalities/provinces across China (see Additional File 1), spanning from January 2012 to December 2021. Neonatal IBIs, including bacteremia and/or bacterial meningitis, are defined as the isolation of pathogens in blood and/or cerebrospinal fluid (CSF) cultures respectively [14]. Each participating hospital was equipped with a level III neonatal intensive care unit (NICU), comprising a minimum of 30 beds [15]. They had advanced laboratory facilities and expertise for treating critically ill newborns. The inclusion criteria of patients were: (1) with manifestations of infections, including fever, grunting, cyanosis, apnea, poor perfusion, slow/no response, lethargy, seizure, and irritability; (2) with positive cultures of blood and/or CSF; (3) samples of blood or CSF cultures were collected within 28 days after birth; (4) gestational age ≧ 37 weeks. The exclusion criteria were: (1) lack of the results for antibiotic susceptibility tests; (2) only contaminating bacteria in blood and/or CSF cultures.

The study was conducted following the principles of the Declaration of Helsinki (1964). The study was approved by the Medical Ethical Committees of each hospital, and subsequent data were shared with other coordinating centers (approval number: XHEC − C−2017 − 084).

### Data collection

Information on pathogens of neonatal IBI cases was extracted from the electronic medical records of each hospital, as well as the corresponding antimicrobial susceptibility data. Minimum inhibitory concentration (MIC) data were collected since 2019, as it was available to most participating hospitals during that time. Other characteristics, including demographic information, age at onset, clinical signs and symptoms, and laboratory data were also recorded in detail.

### Bacterial culture

A uniform method of bacterial culture was used in the participating hospitals. Blood and CSF samples were aseptically inoculated into single aerobic bottles using an automated BacT/ALERT system (BioMérieux, France). As outlined in our previous research, we focused on the four most prevalent pathogens that cause IBIs in full-term neonates, namely Escherichia coli, Group B Streptococcus (GBS), Enterococcus spp., and Staphylococcus aureus [14]. In contrast, organisms such as coagulase-negative staphylococci, Bacillus non-cereus/non-anthracis, diphtheroids, Lactobacillus, Micrococcus, and viridans group streptococci were considered contaminants [16].

### Antibiotic susceptibility test

Antibiotics recommended for infectious conditions of neonates were selected for susceptibility analysis. The laboratories of all participating hospitals used the same method for qualitative determination of antimicrobial susceptibility, which is called the Kirby-Bauer disk diffusion method. MIC (µg/mL), the lowest concentration of antibiotic that inhibited the growth of bacteria, was determined using the agar dilution method recommended by the Clinical and Laboratory Standards Institute (CLSI) [17]. The resistant and intermediate resistant categories were merged, and the ESBL-producing isolates were detected using the double disk synergy test (DDST), as described in the CLSI [17, 18]. The susceptibility of gentamicin for Enterococcus spp. was detected using high-level aminoglycoside resistance testing. The Staphylococcus aureus strains were classified as MRSA according to their oxacillin resistance levels (MIC ≥ 4 µg/mL) [19].

### Statistical analysis

To demonstrate the temporal trend in percentage of susceptibility to relevant antibiotics by strains, we introduced the Joinpoint regression technique, a statistical modeling approach used to identify temporal changes in event rates. Joinpoint program is fitted with a multi-segmented line to achieve a superior fitting than a less segmented line or a single line. Each joinpoint represents a change in the trend with statistical significance. The natural logarithm of incidence rate is fitted into a straight line as the function of calendar year in each segment, thus the estimated annual percentage change (APC) along with its 95% confidence intervals (CIs) are obtained. The slope of each line segment is indicated by APC, showing both gradient and direction. The average annual percentage change (AAPC) is calculated by assuming that there is only one line throughout the study period [20 − 22]. In the process of our Joinpoint regression analysis, the Monte Carlo simulation technique was used to model fitting. A default permutation test was applied to predict the number of joinpoints, and the number of permutations was 4499 after trading off the computer time and the consistency of p-values. Frequencies of categorical variables and medians and interquartile ranges of continuous variables were presented in the results. Mantel-Haenszel linear-by-linear association chi-square test was employed to analyze the trends in the levels of MIC over time by isolates against corresponding antibiotics. Support information can be accessed on the official IBM website (https://www.ibm.com/support/pages/mantel-haenszel-test-trend-available-crosstabs). All tests, including Mantel-Haenszel linear-by-linear association chi-square test and permutation test in the Joinpoint regression, are two-sided. The statistical significance was set at *P* < 0.05. Statistical analyses were performed using SPSS software (Version 26.0, IBM Corp, Armonk, NY, USA) and Joinpoint Regression Program (Version 4.9.1.0, Calverton, Maryland).

## Results

### Participants and pathogen distribution

**Fig. 1:**
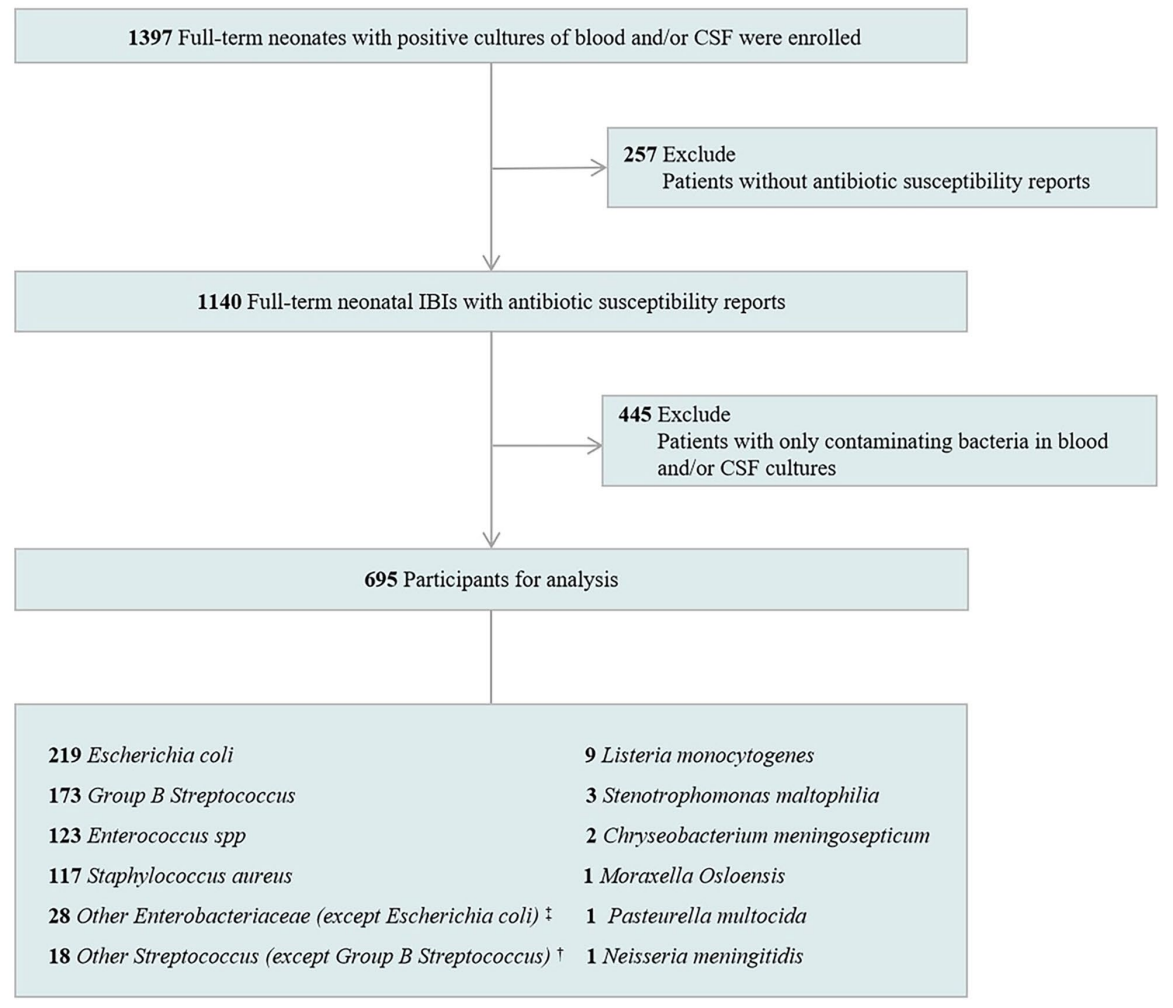
Study subjects flow chart. IBIs, invasive bacterial infections; CSF, cerebrospinal fluid. ^‡^ Including Salmonella species, Proteus species, Enterobacter cloacae, Enterobacter aerogenes, Enterobacter sakazakii, and Enterobacter asburiae. ^†^ Including hemolytic Streptococcus, Streptococcus gallolyticus, Streptococcus constellatus, Streptococcus bradycosa, Streptococcus salivary, Streptococcus pneumoniae, and Streptococcus bovis

A total of 1397 full-term neonates met the inclusion criteria between January 1, 2012 and December 31, 2021. After excluding patients without antibiotic susceptibility reports (*n* = 257, 18.4%) and those with only contaminated strains in the blood or CSF cultures (*n* = 445, 31.9%), 695 episodes of neonatal IBIs were ultimately enrolled in this study, including 197 early-onset cases with age of onset 3 days or less after birth and 498 late-onset cases with age of onset more than 3 days after birth (Fig. 1). Among these 695 cases, 547 (78.7%) had bacteremia without bacterial meningitis, 55 (7.9%) had bacterial meningitis without bacteremia, and 93 (13.4%) had both bacteremia and bacterial meningitis. The yearly demographic characteristics of the patients are described in Table 1, which remained relatively stable over the decade. Male patients constituted 66.3% of the total cases, and vaginal deliveries accounted for 75.5%. Moreover, 68 neonates (9.8%) had invasive procedures like operations, invasive ventilation or central venous catheters. The overall mortality rate of IBIs was 3.7%.

**Table 1:** Annual demographic characteristics of full-term neonates with IBIs

	No. of full-term neonates with IBIs per year									
	2012 (*n* = 47)	2013 (*n* = 50)	2014 (*n* = 69)	2015 (*n* = 75)	2016 (*n* = 98)	2017 (*n* = 64)	2018 (*n* = 73)	2019 (*n* = 74)	2020 (*n* = 65)	2021 (*n* = 80)
Birth weight, median (IQR), g	3300.00 (3000.00–3720.00)	3200 0.00 (3020.00–3600.00)	3307.50 (2825.00–3450.00)	3300.00 (3100.00–3800.00)	3300.00 (3000.00–3565.00)	3300.00 (3050.00–3672.50)	3260.00 (3025.00–3445.00)	3300.00 (3045.00–3545.00)	3475.00 (3185.00–3697.50)	3232.50 (2950.00–3812.50)
Gender, No. (%)										
Male	35 (74.47)	36 (72.00)	41 (59.42)	48 (64.00)	48 (48.98)	48 (75.00)	47 (64.38)	56 (75.68)	49 (75.38)	53 (66.25)
Female	12 (25.53)	14 (28.00)	28 (40.58)	27 (36.00)	50 (51.02)	16 (25.00)	26 (35.62)	18 (24.32)	16 (24.62)	27 (33.75)
Mode of delivery, No. (%)										
Cesarean	10 (21.28)	8 (16.00)	22 (31.88)	15 (20.00)	12 (12.24)	12 (18.75)	20 (27.40)	8 (10.81)	29 (44.62)	34 (42.50)
Vaginal	37 (78.72)	42 (84.00)	47 (68.12)	60 (80.00)	86 (87.76)	52 (81.25)	53 (72.60)	66 (89.19)	36 (55.38)	46 (57.50)
Gestational age, median (IQR), d	280.00 (266.00–284.75)	270.00 (266.00–275.00)	270.50 (266.00–279.75)	276.00 (268.00–281.00)	273.00 (266.00–280.00)	273.50 (269.00–281.50)	275.00 (266.00–281.00)	275.00 (269.00–281.00)	275.50 (272.00–282.75)	274.00 (267.75–279.25)

Abbreviations: IBIs, invasive bacterial infections; IQR, interquartile range

With regard to the pathogens, the top four pathogens accounted for 90.9%, including Escherichia coli (219, 31.5%), GBS (173, 24.9%), Enterococcus spp. (123, 17.7%), and Staphylococcus aureus (117, 16.8%). Other isolates are listed in Fig. 1.

### Trends in antimicrobial susceptibility in neonatal IBIs

**Fig. 2:**
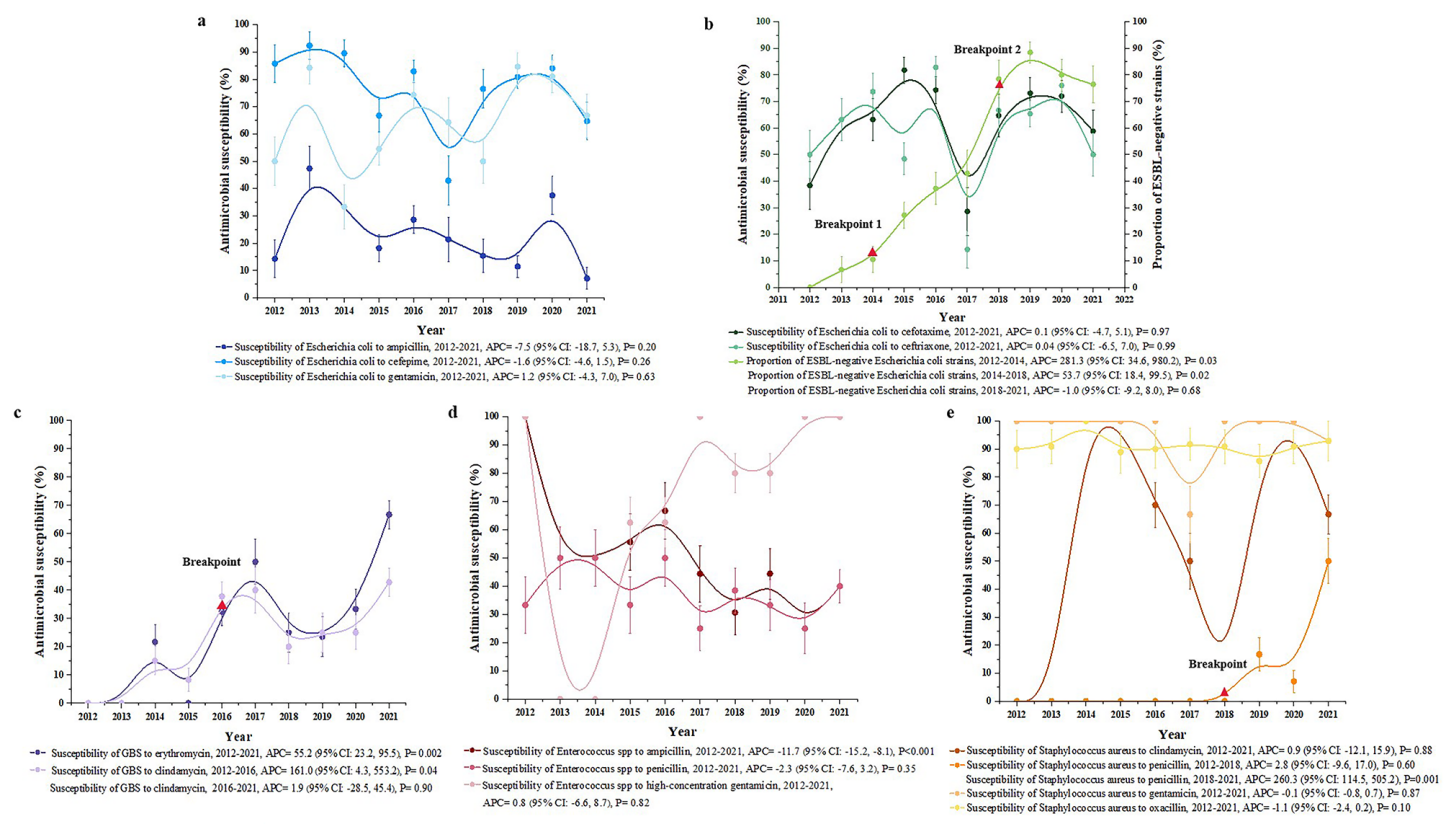
Trends in antimicrobial susceptibility of the most common pathogens in neonatal IBIs, 2012 to 2021. **a** Antimicrobial susceptibility of Escherichia coli; **b** Antimicrobial susceptibility of Escherichia coli and its proportion of ESBL-negative strains; **c** Antimicrobial susceptibility of GBS; **d** Antimicrobial susceptibility of Enterococcus spp.; **e** Antimicrobial susceptibility of Staphylococcus aureus. APC, annual percentage change; GBS, Group B Streptococcus; ESBL, extended-spectrum beta-lactamase; CI, confidence interval. Based on the annual antibiotic susceptibility data of Chinese full-term neonates with IBIs collected in 17 hospitals from 2012 to 2021. Figure 2 exhibits the B-spline fitting curves describing the temporal trends in the prevalence of antimicrobial susceptibility by the four most prevalent pathogens during these 10 years. Data points indicate the proportion of annual susceptible strains. These estimates are presented along with error bars that depict 95% CIs. The breakpoints indicate that the trend of antimicrobial susceptibility varied across different time stages. APC is estimated by Joinpoint regression, which represents the slope of each line segment

**Fig. 3:**
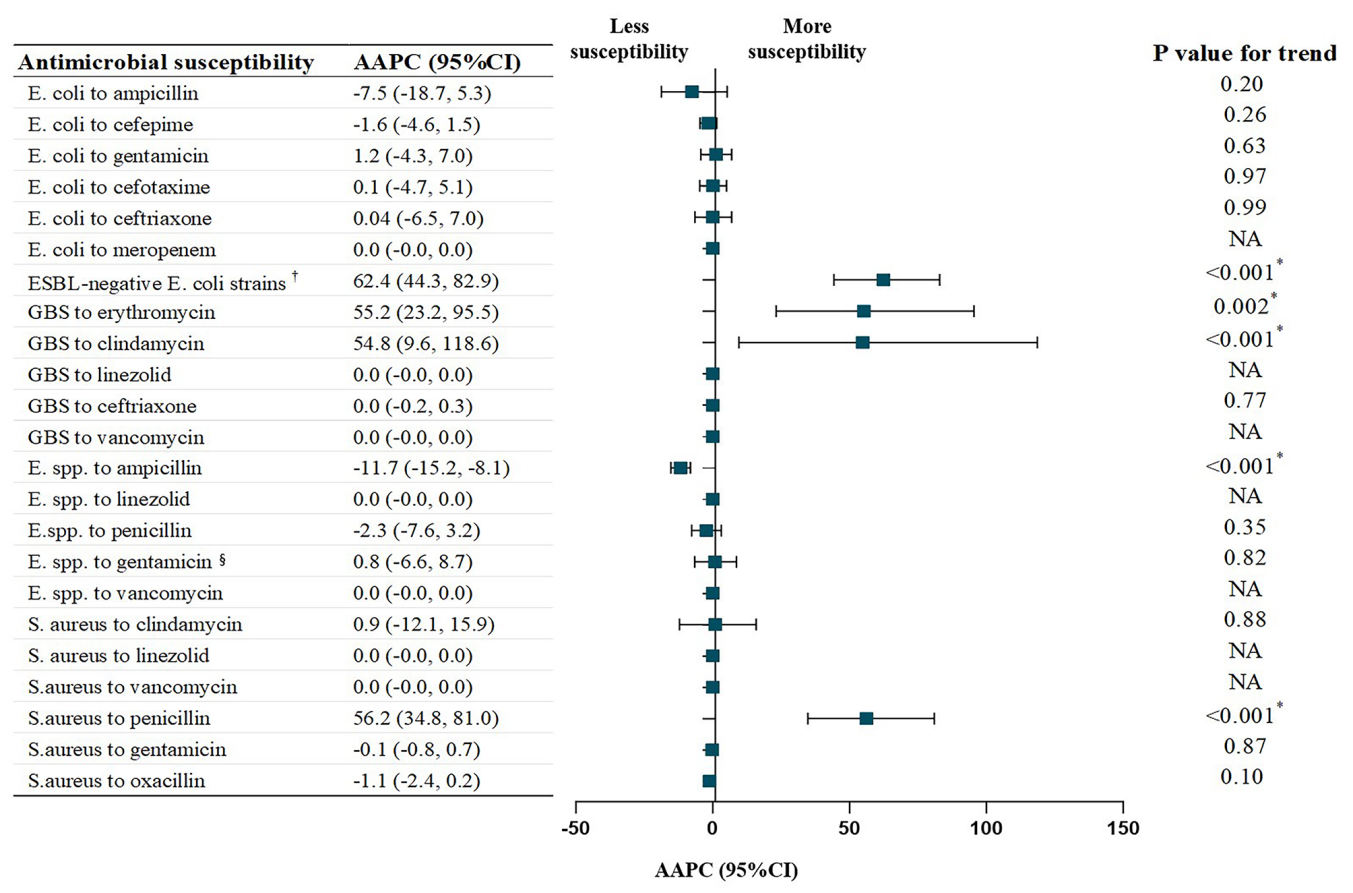
Average annual percentage changes for antimicrobial susceptibility in neonatal IBIs by pathogens, 2012 to 2021. AAPC, average annual percentage change; E. coli, Escherichia coli; GBS, Group B Streptococcus; E. spp., Enterococcus spp.; S. aureus, Staphylococcus aureus; ESBL, extended-spectrum beta-lactamase; CI, confidence interval; NA, not available. Based on the annual antibiotic susceptibility data of Chinese full-term neonates with IBIs collected in 17 hospitals from 2012 to 2021. AAPC represents the estimated mean change in antimicrobial susceptibility of common pathogens in neonatal IBIs from 2012 to 2021, which is calculated by assuming that there are no flection points throughout the study period. ^†^ Proportion of ESBL-negative E. coli strains. ^§^ Enterococcus spp. to high-concentration gentamicin. ^*^ Significant at *p* < 0.05

Figure 2 exhibits the B-spline fitting curves describing the temporal trends in the prevalence of antimicrobial susceptibility by the four most prevalent pathogens during the past 10 years and highlighting the inflection points with statistical significance. The annual proportions of susceptible strains and their corresponding 95% CIs are shown in Additional File 2. Figure 3 illustrates the AAPC in antibiotic susceptibility of four common pathogens in neonatal IBIs from 2012 to 2021, which is calculated by assuming that there are no flection points throughout the study period. The findings revealed that there was no significant change observed in the antibiotic susceptibility of gram-negative bacilli. However, the proportion of ESBL-negative Escherichia coli strains increased significantly (AAPC = 62.4%; 95%CI, 44.3–82.9%; P for trend < 0.001). Among gram-positive cocci, GBS and Staphylococcus aureus appeared to have pronounced improvement in some antimicrobial susceptibilities over the past decade, such as GBS to erythromycin (AAPC = 55.2%; 95%CI, 23.2–95.5%; P for trend = 0.002), GBS to clindamycin (AAPC = 54.8%; 95%CI, 9.6–118.6%; P for trend < 0.001), and Staphylococcus aureus to penicillin (AAPC = 56.2%; 95%CI, 34.8–81.0%; P for trend < 0.001). However, Enterococcus spp. showed a declining trend to ampicillin (AAPC = − 11.7%; 95%CI, − 15.2% to − 8.1%; P for trend < 0.001) (Fig. 3).

Changes in the prevalence of susceptibility to antibiotics, however, are not constant over time. Individually, for Escherichia coli, except meropenem which maintained almost 100% sensitivity, the susceptibility to third- or fourth-generation cephalosporins and gentamicin remained relatively stable, whereas ampicillin displayed a slight decrease (Fig. 2a). Nevertheless, the rate of ESBL-negative strains increased significantly and persistently, especially during the periods of 2012–2014 (APC = 281.3%; 95% CI, 34.6–980.2%; P for trend = 0.03) and 2014–2018 (APC = 53.7%; 95% CI, 18.4–99.5%; P for trend = 0.02). After 2018, the prevalence stabilized within the range of 76.5–88.5% (APC = − 1.0%; 95% CI, − 9.2–8.0%; P for trend = 0.68) (Fig. 2b). For gram-positive cocci, the improvement in susceptibility of GBS to clindamycin exhibited a subsequent decrease after 2016, with fluctuations ranging from 20.0 to 42.8% between 2016 and 2021 (APC = 1.9%; 95% CI, − 28.5–45.4%; P for trend = 0.90) (Fig. 2c). The sensitivity of Enterococcus spp. to penicillin and high-concentration gentamicin did not change significantly; however, its susceptibility to ampicillin gradually declined over time, from 100.0 to 25.0% (APC = − 11.7%; 95% CI, − 15.2% to − 8.1%; P for trend < 0.001) (Fig. 2d). Regarding Staphylococcus aureus, a substantial susceptibility improvement was only observed in penicillin with an inflection point in 2018. Before 2018, the strain exhibited a complete resistance with the prevalence of susceptibility close to 0.0%; while after that, there was a notable advancement, reaching 50% by 2021 (APC = 260.3%; 95% CI, 114.5–505.2%; P for trend = 0.001). Meanwhile, Staphylococcus aureus also displayed a high sensitivity to gentamicin throughout this decade, except for the sporadic emergence of resistant strains in 2017 and 2021. The prevalence of MRSA was comparatively low, as the majority of subjects in our study were community-acquired infections. The annual susceptibility of Staphylococcus aureus to oxacillin primarily fluctuated from 85.7 to 92.9% (Fig. 2e). In addition, it was reassuring that all gram-positive cocci, GBS, Enterococcus spp., and Staphylococcus aureus were shown sustained susceptibility to linezolid and vancomycin throughout the past decade. Over the ten years of this study, we observed that the transition period was around 2014 to 2018. Detailed model outputs of Joinpoint regression are shown in Additional File 3.

### Changes in antibiotic MICs in neonatal IBIs

**Fig. 4:**
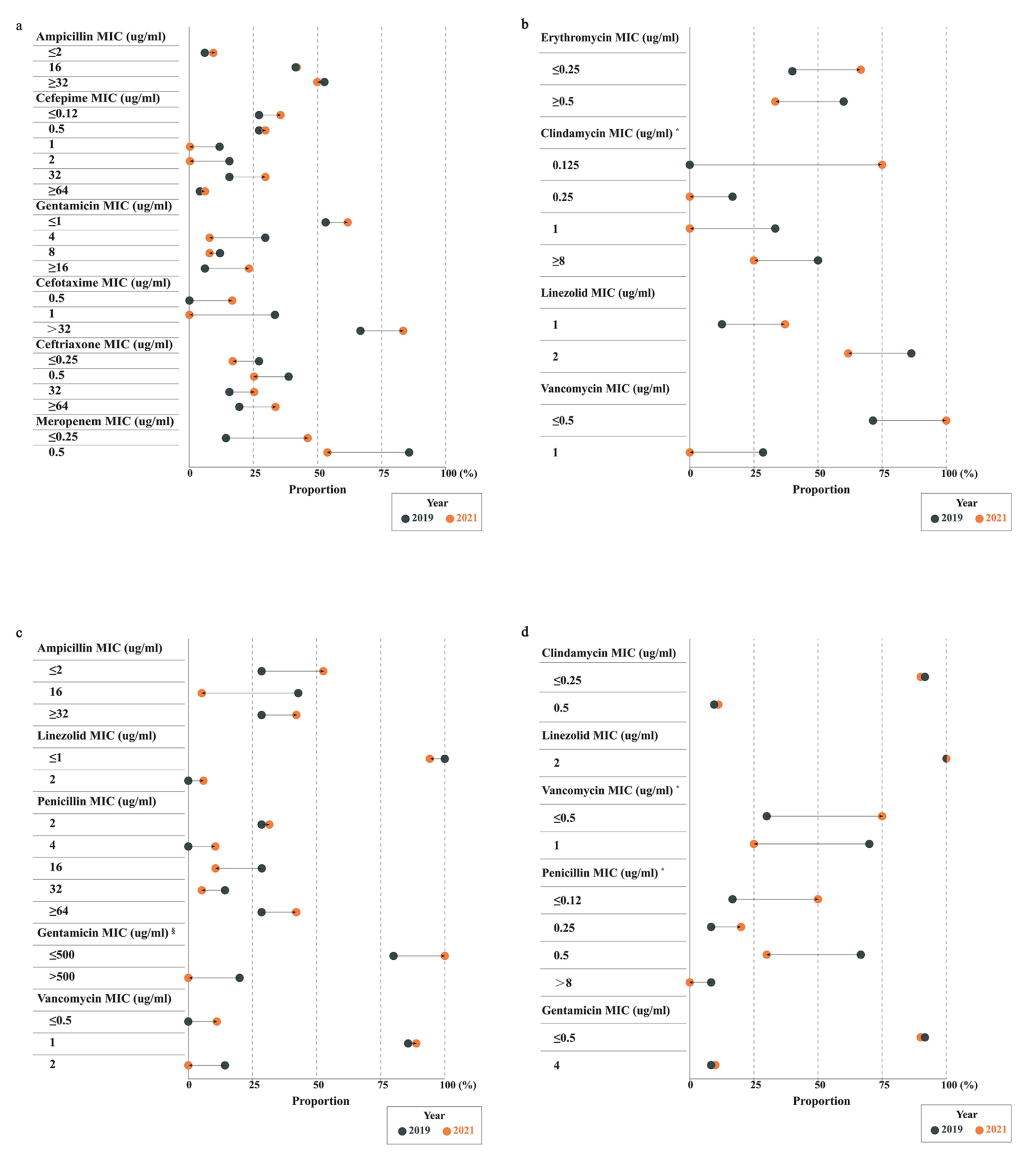
Changes in MICs of antibiotics against pathogens in neonatal IBIs, 2019 to 2021. **a** The MIC of antibiotics against Escherichia coli; **b** The MIC of antibiotics against GBS; **c** The MIC of antibiotics against Enterococcus spp.; **d** The MIC of antibiotics against Staphylococcus aureus. MIC, Minimum inhibitory concentration; GBS, Group B Streptococcus. Based on data of MIC for antibiotics against common pathogens in neonatal IBIs collected in 17 Chinese hospitals from 2019 to 2021. Figure 4 exhibits the changes in antibiotic MICs between 2019 and 2021. The arrows pointing to the right represent increases in proportion over time, while the arrows pointing to the left indicate decreases. If the proportion of a strain with low MIC level increased in a certain pathogen, it theoretically indicated that the susceptibility of the corresponding antibiotic to this pathogen improved during the three years, and vice versa. ^§^ High-concentration gentamicin MIC (ug/ml). ^*^ Mantel-Haenszel linear-by-linear association chi-square test revealed a notable p for trend (*p* < 0.05)

To obtain a more precise profile of subtle changes in susceptibility of pathogens to antibiotics in neonates, we further observed the changes in antibiotic MICs between 2019 and 2021 (Fig. 4). The arrows pointing to the right exhibit increases in proportion over time, while arrows pointing to the left represent decreases. Compared with 2019, the proportion of strains with relatively low MIC levels in some pathogens increased to varying degrees in 2021. Specifically, in sensitive strains of GBS, the percentages of the strains with low MIC values for erythromycin, clindamycin, linezolid, and vancomycin increased in 2021, but only GBS to clindamycin with a significant linear trend (*P* = 0.016), consistent with the observed trend analysis (Fig. 4b and Additional File 4). Regarding Enterococcus spp., although the trend analysis above showed a decline in ampicillin sensitivity, there was no significant change in the MIC from 2019 to 2021 (Fig. 4c). In terms of MIC values for antimicrobials to Staphylococcus aureus strains, the proportion of MIC ≤ 0.12 µg/ml for penicillin tripled in 2021 compared with 2019 (50.0% vs. 16.7%), with statistical significance (*P* = 0.033). Although Staphylococcus aureus showed sustained susceptibility to vancomycin in these years, its proportion of relatively low MIC levels still increased in a linear trend (*P* = 0.035) (Fig. 4d and Additional File 4). Finally, in gram-negative bacteria, Escherichia coli strain did not show any obvious improvement in the composition ratio of MIC values for the antibiotics such as ampicillin, cefepime, cefotaxime, ceftriaxone, and gentamicin over the study period.

## Discussion

Our study explored the dynamics of pathogen susceptibility to antibiotics among full-term neonates with IBIs in 17 facilities across China from 2012 to 2021. During the decade, there was a rapid increase in the percentage of ESBL-negative Escherichia coli, reaching approximately 80%, which approached the level observed in the United States (93 − 95%) [23]. The susceptibility of pathogens to most antibiotics generally tended to be stable or improved over various periods, except for Enterococcus spp. to ampicillin. Specifically, the susceptibility of GBS to erythromycin and clindamycin improved after 2012; however, it still fell behind the levels in North American countries (0.0 − 66.7% vs. 50 − 84%; 0.0 − 42.8% vs. 58 − 95%, respectively) [24]. Following the release of a refined management of antimicrobials for children in 2018, a breakthrough improvement was observed in the susceptibility of Staphylococcus aureus to penicillin. The proportion of lower MICs for certain antibiotics appeared to have improved from 2019 to 2021, in which GBS demonstrated prominent improvement in its susceptibility to clindamycin, as did Staphylococcus aureus to penicillin and vancomycin.

The increased antimicrobial susceptibility can be attributed to several factors, one of which was the implementation of significant reforms in antibiotic manufacturing, circulation, and utilization by the Chinese government in early years. It was exemplified by the Administrative Measures for Clinical Application of Antibiotics issued by the Ministry of Health in China in 2012. This policy primarily aimed to prohibit the over-the-counter sales of antibiotics and mitigate antimicrobial abuse rates [25]. By 2016, the 5-year National Action Plan was enacted, including enhancing drug-resistant strain monitoring systems, prohibiting routine administration of antibiotics to healthy livestock, and intensifying prevention measures against environmental pollution caused by antibiotics [26]. Subsequently, in 2018, the National Health Commission further issued a notice on strengthening the clinical management of antibiotics in vulnerable populations such as children [27]. In response, all clinicians in our participating hospitals followed the antibiotic prescription patterns among Chinese children [28] and executed various strategies, including implementing broad-spectrum antibiotic prescription authorization, limitations on the number of prescribed medicines, regular evaluation of antibiotics use density, and real-time monitoring of antimicrobial resistance data by pharmacists. Subsequently, we observed a decline in the prevalence of gram-negative bacilli-producing ESBL enzymes, whereas the susceptibility of certain gram-positive cocci to antibiotics improved gradually. Nevertheless, the emergence of each breakpoint in the trend may be attributed to the combined effect of multiple policies, which required a certain period of effort to show up. Therefore, it cannot be explained by any specific policy.

Besides that, another possible contributing factor was the standard therapeutic scheme for certain infectious diseases. Recently, domestic experts have developed several clinical guidelines for antimicrobial use and continuously updated them in real time [29 − 31]. These guidelines were developed with reference to relevant foreign guidelines or protocols [32, 33], and updated based on evidence-based recommendations that align with Chinese population characteristics. One significant initiative was to gradually implement prenatal screening for GBS and utilize penicillin or ampicillin as first-line intrapartum antimicrobial prophylaxis in many Chinese hospitals, aiming to standardize prenatal antibiotic use [34, 35]. The administration of this standardized treatment regimen did contribute to reducing the irrational use of antibiotics and enhancing the susceptibility of GBS to erythromycin and clindamycin, as evidenced by our research findings.

Additionally, the coronavirus disease 2019 (COVID − 19) pandemic may also have an impact on public health and the management of diverse healthcare matters [36]. During this epidemic, implementing non-pharmaceutical public health interventions (NPIs), such as strict adherence to isolation protocols, the reinforcement of personal protective measures, the promotion of hand hygiene, and the enhancement of environmental disinfection procedures, were pivotal in curbing nosocomial infections and reducing antibiotic utilization rates. It had been reported that NPIs likely contributed to the decline in invasive bacterial disease incidence in the United States in 2020 [37], and caused reductions in antibiotic prescribing among children [38]. As shown in our study, the increase in the proportion of GBS strains with relatively low MIC value of clindamycin in 2021 was obvious compared with 2019, as well as Staphylococcus aureus to penicillin. Furthermore, although the antibiotic susceptibility test indicated that some gram-positive cocci consistently remained sensitive to vancomycin and linezolid, there was still a discernible increase in the proportion of strains with lower MIC levels. All these improvements in antimicrobial susceptibility may be partially attributed to the modifications above in medical models and health concepts during the epidemic period.

Our study had several limitations. First, although this was a multicenter survey, our study was not designed as a national census. Due to the unbalanced regional distribution of participating hospitals, it may not reflect the specific situation of neonatal antimicrobial resistance in different regions of China. Second, the contamination rate in this study was relatively high. This suggested that there was still some space for improvement in the prior sample collection process. On the other hand, it also probably reflected the inherent challenges in obtaining high-quality data for neonatal IBIs. Third, given that the ESBL testing was not routinely conducted for all Escherichia coli strains, it resulted in a disconnect between ESBL production and third-generation cephalosporin resistance in the early years. Fourth, since the MIC of antibiotics was not determined before 2019, we could only explore its changes over the following three years. Lastly, preterm infants were excluded from the study. Preterm newborns may have a distinct spectrum of pathogens compared with full-term neonates and are more vulnerable to drug-resistant bacterial infections due to prolonged hospitalization in NICUs and exposure to more invasive procedures. This could potentially obscure the true trend of antimicrobial susceptibility if they are included in the study.

## Conclusions

In this study, antimicrobial susceptibility in full-term neonates with IBIs in China appeared to be improved and stabilized for most pathogens between 2012 and 2021, implying that the effectiveness of principles and practice of antibiotic stewardship had gradually emerged. The current domestic situation, however, still remains severe compared with that in developed countries [39]. Therefore, in addition to the further improvements in hospital capacity of infection control, individualized management should be accelerated to promote targeted care for vulnerable infants. Our multicenter study may help decision-makers to institute sustainable antimicrobial strategies.

### Electronic supplementary material

Below is the link to the electronic supplementary material.

Additional File 1. Table of participating hospitals in this study

Additional File 2. Table of trends in the antimicrobial susceptibility of common pathogens among Chinese full-term neonates with IBIs

Additional File 3. Table of detailed model outputs of Joinpoint regression

Additional File 4. Table of Mantel-Haenszel linear-by-linear chi-square test for MIC of antibiotics against pathogens in Chinese full-term neonates with IBIs, 2019–2021

## Data Availability

All data generated or analyzed in the study are included within this article and its supplementary information files.

## Abbreviations

AAPC: Average annual percentage change
APC: Annual percentage change
CI: Confidence interval
CLSI: Clinical and Laboratory Standards Institute
COVID − 19: Coronavirus disease 2019
CSF: Cerebrospinal fluid
DDST: Double disk synergy test
ESBL: Extended-spectrum beta-lactamase
E. coli: Escherichia coli
E. spp.: Enterococcus spp
GBS: Group B Streptococcus
IBIs: Invasive bacterial infections
IQR: Interquartile range
MICs: Minimum inhibitory concentrations
MRSA: Methicillin-resistant Staphylococcus aureus
NPIs: Non-pharmaceutical public health interventions
S. aureus: Staphylococcus aureus
