# Supplementary material for: Trends in the antimicrobial susceptibility among Chinese neonates from 2012 to 2021: a multicenter study
Source: Antimicrob Resist Infect Control. 2024 Jul 30;13:83. doi: 10.1186/s13756-024-01440-2 (PMC11290293; doi:10.1186/s13756-024-01440-2)
Supplement: Supplementary file 1 — Additional File 1. Table of participating hospitals in this study [file 13756_2024_1440_MOESM1_ESM.pdf]

Additional file 1

Table: Participating hospitals in this study

Province/municipality	Participating hospitals
Shanghai	Xinhua Hospital
	Children's Hospital of Shanghai
	Shanghai Children's Medical Center
	Children's Hospital of Fudan University
	Obstetrics & Gynecology Hospital of Fudan University
	International Peace Maternity and Child Health Hospital
Jiangsu Province	Children's Hospital of Nanjing Medical University
	Affiliated Women's Hospital of Jiangnan University
	Changzhou Maternal and Child Health Care Hospital
	Affiliated Hospital of Jiangsu University
	Affiliated Hospital of Yangzhou University
Zhejiang Province	Jiaxing University Affiliated Women and Children Hospital
Henan Province	First Affiliated Hospital of Zhengzhou University
Shandong Province	The Affiliated Hospital of Qingdao University
Guangdong Province	Dongguan Children's Hospital Affiliated to Guangdong Medical University
Sichuan Province	The Affiliated Hospital of Southwest Medical University
Liaoning Province	Shengjing Hospital of China Medical University
