# Supplementary material for: Trends in the antimicrobial susceptibility among Chinese neonates from 2012 to 2021: a multicenter study
Source: Antimicrob Resist Infect Control. 2024 Jul 30;13:83. doi: 10.1186/s13756-024-01440-2 (PMC11290293; doi:10.1186/s13756-024-01440-2)
Supplement: Supplementary file 2 — Additional File 2. Table of trends in the antimicrobial susceptibility of common pathogens among Chinese full-term neonates with IBIs [file 13756_2024_1440_MOESM2_ESM.pdf]

Additional file 2

Table: Trends in the antimicrobial susceptibility of common pathogens among Chinese full-term neonates with IBIs

	Annual susceptible strains, % (95% CI)										P value for trend [b]
	2012	2013	2014	2015	2016	2017	2018	2019	2020	2021	
E. coli	n=14	n=19	n=19	n=33	n=35	n=14	n=17	n=26	n=25	n=17	
Ampicillin	14.3 (7.5, 21.1)	47.4 (39.3, 55.5)	33.3 (25.4, 41.2) [a]	18.2 (13.3, 23.1)	28.6 (23.4, 33.8)	21.5 (13.6, 29.4)	15.4 (9.3, 21.5) [a]	11.5 (7.3, 15.7)	37.5 (30.7, 44.3) [a]	7.1 (3.2, 11.0) [a]	0.20
Cefepime	85.7 (78.8, 92.6)	92.3 (87.1, 97.5) [a]	89.5 (84.4, 94.6)	66.7 (60.5, 72.9)	82.9 (78.6, 87.2)	42.9 (34.1, 51.7)	76.5 (69.4, 83.6)	80.8 (76.6, 85.0)	84.0 (78.9, 89.1)	64.7 (57.8, 71.6)	0.26
Gentamicin	50.0 (41.2, 58.8)	84.2 (78.1, 90.3)	33.3 (25.4, 41.2) [a]	54.5 (48.6, 60.4)	74.3 (69.1, 79.5)	64.3 (55.4, 73.2)	50.0 (42.1, 57.9) [a]	84.6 (79.5, 89.7)	81.0 (75.1, 86.9) [a]	66.7 (58.9, 74.5) [a]	0.63
Cefotaxime	38.4 (29.6, 47.2) [a]	63.2 (55.1, 71.3)	63.2 (55.1, 71.3)	81.8 (76.7, 86.9)	74.3 (69.4, 79.2)	28.6 (19.7, 37.5)	64.7 (56.5, 72.9)	73.1 (67.2, 79.0)	72.0 (66.2, 77.8)	58.8 (50.7, 66.9)	0.97
Ceftriaxone	50.0 (41.1, 58.9)	63.2 (55.1, 71.3)	73.7 (66.6, 80.8)	48.4 (42.5, 54.3)	82.9 (78.6, 87.2)	14.3 (7.1, 21.5)	66.7 (58.8, 74.6) [a]	65.4 (60.3, 70.5)	76.0 (70.2, 81.8)	50.0 (42.1, 57.9) [a]	0.99
Meropenem	100.0 (100.0, 100.0)	100.0 (100.0, 100.0)	100.0 (100.0, 100.0)	100.0 (100.0, 100.0)	100.0 (100.0, 100.0)	100.0 (100.0, 100.0)	100.0 (100.0, 100.0)	100.0 (100.0, 100.0)	100.0 (100.0, 100.0)	100.0 (100.0, 100.0)	NA
ESBL-negative strains [c]	0.0 (0.0, 0.0) [a]	6.7 (1.8, 11.6) [a]	10.5 (5.4, 15.6)	27.2 (22.0, 32.4)	37.2 (31.3, 43.1)	42.9 (34.1, 51.7)	78.5 (71.4, 85.6) [a]	88.5 (84.3, 92.7)	80.0 (74.2, 85.8)	76.5 (69.6, 83.4)	<0.001 [d]

Table: *Trends in the antimicrobial susceptibility of common pathogens among Chinese full-term neonates with IBIs (continued)*

	Annual susceptible strains, % (95% CI)										P value for trend [b]
	2012	2013	2014	2015	2016	2017	2018	2019	2020	2021	
GBS	n=11	n=10	n=23	n=20	n=37	n=20	n=20	n=17	n=6	n=9	
Erythromycin	0.0 (0.0, 0.0)	0.0 (0.0, 0.0)	21.7 (15.8, 27.6)	0.0 (0.0, 0.0)	32.4 (27.3, 37.5)	50.0 (42.2, 57.8)	25.0 (17.9, 32.1)	23.5 (16.6, 30.4)	33.3 (26.5, 40.1)	66.7 (61.6, 71.8)	0.002 [d]
Clindamycin	0.0 (0.0, 0.0)	0.0 (0.0, 0.0)	15.0 (10.1, 19.9) [a]	8.3 (4.1, 12.5) [a]	37.8 (32.7, 42.9)	40.0 (32.2, 47.8)	20.0 (13.9, 26.1)	25.0 (18.1, 31.9) [a]	25.0 (18.8, 31.2) [a]	42.8 (37.7, 47.9) [a]	<0.001 [d]
Linezolid	100.0 (100.0, 100.0)	100.0 (100.0, 100.0)	100.0 (100.0, 100.0)	100.0 (100.0, 100.0)	100.0 (100.0, 100.0)	100.0 (100.0, 100.0)	100.0 (100.0, 100.0)	100.0 (100.0, 100.0)	100.0 (100.0, 100.0)	100.0 (100.0, 100.0)	NA
Ceftriaxone	100.0 (100.0, 100.0)	100.0 (100.0, 100.0)	91.3 (87.9, 94.7)	100.0 (100.0, 100.0)	100.0 (100.0, 100.0)	100.0 (100.0, 100.0)	100.0 (100.0, 100.0)	100.0 (100.0, 100.0)	100.0 (100.0, 100.0)	100.0 (100.0, 100.0)	0.77
Vancomycin	100.0 (100.0, 100.0)	100.0 (100.0, 100.0)	100.0 (100.0, 100.0)	100.0 (100.0, 100.0)	100.0 (100.0, 100.0)	100.0 (100.0, 100.0)	100.0 (100.0, 100.0)	100.0 (100.0, 100.0)	100.0 (100.0, 100.0)	100.0 (100.0, 100.0)	NA
E. Spp.	n=12	n=10	n=12	n=12	n=12	n=9	n=13	n=10	n=8	n=25	
Ampicillin	100.0 (100.0, 100.0) [a]	50.0 (38.8, 61.2)	50.0 (39.8, 60.2)	55.6 (45.7, 65.5) [a]	66.7 (57.1, 76.3)	44.4 (34.6, 54.2)	30.7 (22.8, 38.6)	44.4 (35.5, 53.3) [a]	25.0 (15.9, 34.1)	40.0 (33.8, 46.2)	<0.001 [d]
Linezolid	100.0 (100.0, 100.0)	100.0 (100.0, 100.0)	100.0 (100.0, 100.0)	100.0 (100.0, 100.0)	100.0 (100.0, 100.0)	100.0 (100.0, 100.0)	100.0 (100.0, 100.0)	100.0 (100.0, 100.0)	100.0 (100.0, 100.0)	100.0 (100.0, 100.0)	NA

Table: Trends in the antimicrobial susceptibility of common pathogens among Chinese full-term neonates with IBIs (continued)

	Annual susceptible strains, % (95% CI)										P value for trend [b]
	2012	2013	2014	2015	2016	2017	2018	2019	2020	2021	
E. Spp.	n=12	n=10	n=12	n=12	n=12	n=9	n=13	n=10	n=8	n=25	
Penicillin	33.3 (23.7, 42.9)	50.0 (38.8, 61.2)	50.0 (39.8, 60.2)	33.3 (23.7, 42.9)	50.0 (39.8, 60.2)	25.0 (16.9, 33.1) [a]	38.5 (30.6, 46.4)	33.3 (24.4, 42.2) [a]	25.0 (16.2, 33.8)	40.0 (33.9, 46.1)	0.35
High-concentration gentamicin	100.0 (100.0, 100.0) [a]	0.0 (0.0, 0.0)	0.0 (0.0, 0.0) [a]	62.5 (53.6, 71.4) [a]	62.5 (53.6, 71.4) [a]	100.0 (100.0, 100.0)	80.0 (72.9, 87.1) [a]	80.0 (72.9, 87.1)	100.0 (100.0, 100.0)	100.0 (100.0, 100.0)	0.82
Vancomycin	100.0 (100.0, 100.0)	100.0 (100.0, 100.0)	100.0 (100.0, 100.0)	100.0 (100.0, 100.0)	100.0 (100.0, 100.0)	100.0 (100.0, 100.0)	100.0 (100.0, 100.0)	100.0 (100.0, 100.0)	100.0 (100.0, 100.0)	100.0 (100.0, 100.0)	NA
S. aureus	n=10	n=11	n=10	n=9	n=10	n=12	n=12	n=14	n=14	n=15	
Clindamycin	0.0 (0.0, 0.0)	0.0 (0.0, 0.0) [a]	100.0 (100.0, 100.0) [a]	100.0 (100.0, 100.0) [a]	70.0 (62.2, 77.8)	50.0 (39.8, 60.2)	0.0 (0.0, 0.0) [a]	85.7 (79.6, 91.8)	100.0 (100.0, 100.0) [a]	66.7 (59.8, 73.6)	0.88
Linezolid	100.0 (100.0, 100.0)	100.0 (100.0, 100.0)	100.0 (100.0, 100.0)	100.0 (100.0, 100.0)	100.0 (100.0, 100.0)	100.0 (100.0, 100.0)	100.0 (100.0, 100.0)	100.0 (100.0, 100.0)	100.0 (100.0, 100.0)	100.0 (100.0, 100.0)	NA
Vancomycin	100.0 (100.0, 100.0)	100.0 (100.0, 100.0)	100.0 (100.0, 100.0)	100.0 (100.0, 100.0)	100.0 (100.0, 100.0)	100.0 (100.0, 100.0)	100.0 (100.0, 100.0)	100.0 (100.0, 100.0)	100.0 (100.0, 100.0)	100.0 (100.0, 100.0)	NA
Penicillin	0.0 (0.0, 0.0)	0.0 (0.0, 0.0) [a]	0.0 (0.0, 0.0)	0.0 (0.0, 0.0)	0.0 (0.0, 0.0)	0.0 (0.0, 0.0) [a]	0.0 (0.0, 0.0)	16.7 (10.6, 22.8) [a]	7.1 (3.0, 11.2)	50.0 (42.1, 57.9) [a]	<0.001 [d]

Table: Trends in the antimicrobial susceptibility of common pathogens among Chinese full-term neonates with IBIs (continued)

	Annual susceptible strains, % (95% CI)										P value for trend [b]
	2012	2013	2014	2015	2016	2017	2018	2019	2020	2021	
S. aureus	n=10	n=11	n=10	n=9	n=10	n=12	n=12	n=14	n=14	n=15	
Gentamicin	100.0 (100.0, 100.0)	100.0 (100.0, 100.0)	100.0 (100.0, 100.0)	100.0 (100.0, 100.0)	100.0 (100.0, 100.0)	66.7 (57.1, 76.3)	100.0 (100.0, 100.0)	100.0 (100.0, 100.0)	100.0 (100.0, 100.0)	92.9 (85.9, 99.9) [a]	0.87
Oxacillin	90.0 (83.3, 96.7)	90.9 (84.8, 97.0)	100.0 (100.0, 100.0) [a]	88.9 (81.5, 96.3)	90.0 (83.3, 96.7)	91.7 (86.0, 97.3)	90.9 (84.8, 97.0) [a]	85.7 (79.6, 91.8)	90.9 (84.8, 97.0) [a]	92.9 (85.9, 99.9) [a]	0.10

Abbreviations: CI, confidence interval; E. coli, Escherichia coli; ESBL, extended-spectrum beta-lactamase; GBS, Group B Streptococcus; E. spp., Enterococcus spp.; S. aureus, Staphylococcus aureus; NA, not available

[a] Our antibiotic susceptibility rate was limited to strains with available drug susceptibility results, as a minority of strains lacked comprehensive susceptibility data for each antibiotic.

[b] The P value of average annual percentage change was calculated using the Joinpoint regression technique.

[c] Proportion of ESBL-negative E. coli strains.

[d] $P < 0.05$.
