# Supplementary material for: Trends in the antimicrobial susceptibility among Chinese neonates from 2012 to 2021: a multicenter study
Source: Antimicrob Resist Infect Control. 2024 Jul 30;13:83. doi: 10.1186/s13756-024-01440-2 (PMC11290293; doi:10.1186/s13756-024-01440-2)
Supplement: Supplementary file 3 — Additional File 3. Table of detailed model outputs of Joinpoint regression [file 13756_2024_1440_MOESM3_ESM.pdf]

## Additional file 3

*Table: Detailed model outputs of Joinpoint regression*

| Antimicrobial susceptibility | Periods (year) | APC (95% CI) | β | t | P | AAPC (95% CI) | β | t/Z [a] | P |
|---|---|---|---|---|---|---|---|---|---|
| E. coli to ampicillin | 2012-2021 | -7.5 (-18.7, 5.3) | -0.08 | -1.39 | 0.20 | -7.5 (-18.7, 5.3) | -0.08 | -1.39 | 0.20 |
| E. coli to cefepime | 2012-2021 | -1.6 (-4.6, 1.5) | -0.02 | -1.21 | 0.26 | -1.6 (-4.6, 1.5) | -0.02 | -1.21 | 0.26 |
| E. coli to gentamicin | 2012-2021 | 1.2 (-4.3, 7.0) | 0.01 | 0.50 | 0.63 | 1.2 (-4.3, 7.0) | 0.01 | 0.50 | 0.63 |
| E. coli to cefotaxime | 2012-2021 | 0.1 (-4.7, 5.1) | 0.0007 | 0.04 | 0.97 | 0.1 (-4.7, 5.1) | 0.0007 | 0.04 | 0.97 |
| E. coli to ceftriaxone | 2012-2021 | 0.04 (-6.5, 7.0) | 0.0004 | 0.01 | 0.99 | 0.04 (-6.5, 7.0) | 0.0004 | 0.01 | 0.99 |
| E. coli to meropenem | 2012-2021 | 0.0 (-0.0, 0.0) | 0.00 | NA | NA | 0.0 (-0.0, 0.0) | 0.00 | NA | NA |
| ESBL-negative E. coli strains [b] | 2012-2014 | 281.3 (34.6, 980.2) | 1.34 | 5.53 | 0.03 [c] | 62.4 (44.3, 82.9) | 0.41 | 8.00 | <0.001 [c] |
| | 2014-2018 | 53.7 (18.4, 99.5) | 0.43 | 7.09 | 0.02 [c] | | | | |
| | 2018-2021 | -1.0 (-9.2, 8.0) | -0.01 | -0.48 | 0.68 | | | | |
| GBS to erythromycin | 2012-2021 | 55.2 (23.2, 95.5) | 0.44 | 4.39 | 0.002 [c] | 55.2 (23.2, 95.5) | 0.44 | 4.39 | 0.002 [c] |
| GBS to clindamycin | 2012-2016 | 161.0 (4.3, 553.2) | 0.96 | 2.69 | 0.04 [c] | 54.8 (9.6, 118.6) | 0.39 | 2.50 | <0.001 [c] |
| | 2016-2021 | 1.9 (-28.5, 45.4) | 0.02 | 0.14 | 0.90 | | | | |
| GBS to linezolid | 2012-2021 | 0.0 (-0.0, 0.0) | 0.00 | NA | NA | 0.0 (-0.0, 0.0) | 0.00 | NA | NA |
| GBS to ceftriaxone | 2012-2021 | 0.0 (-0.2, 0.3) | 0.0003 | 0.30 | 0.77 | 0.0 (-0.2, 0.3) | 0.0003 | 0.30 | 0.77 |
| GBS to vancomycin | 2012-2021 | 0.0 (-0.0, 0.0) | 0.00 | NA | NA | 0.0 (-0.0, 0.0) | 0.00 | NA | NA |
| E. spp. to ampicillin | 2012-2021 | -11.7 (-15.2, -8.1) | -0.12 | -7.16 | <0.001 [c] | -11.7 (-15.2, -8.1) | -0.12 | -7.16 | <0.001 [c] |
| E. spp. to linezolid | 2012-2021 | 0.0 (-0.0, 0.0) | 0.00 | NA | NA | 0.0 (-0.0, 0.0) | 0.00 | NA | NA |
| E. spp. to penicillin | 2012-2021 | -2.3 (-7.6, 3.2) | -0.02 | -0.997 | 0.35 | -2.3 (-7.6, 3.2) | -0.02 | -0.997 | 0.35 |
| E. spp. to high-concentration gentamicin | 2012-2021 | 0.8 (-6.6, 8.7) | 0.01 | 0.23 | 0.82 | 0.8 (-6.6, 8.7) | 0.01 | 0.23 | 0.82 |
| E. spp. to vancomycin | 2012-2021 | 0.0 (-0.0, 0.0) | 0.00 | NA | NA | 0.0 (-0.0, 0.0) | 0.00 | NA | NA |
| S. aureus to clindamycin | 2012-2021 | 0.9 (-12.1, 15.9) | 0.01 | 0.16 | 0.88 | 0.9 (-12.1, 15.9) | 0.01 | 0.16 | 0.88 |

*Table: Detailed model outputs of Joinpoint regression (continued)*

| Antimicrobial susceptibility | Periods (year) | APC (95% CI) | β | t | P | AAPC (95% CI) | β | t/Z [a] | P |
|---|---|---|---|---|---|---|---|---|---|
| S. aureus to linezolid | 2012-2021 | 0.0 (-0.0, 0.0) | 0.00 | NA | NA | 0.0 (-0.0, 0.0) | 0.00 | NA | NA |
| S. aureus to vancomycin | 2012-2021 | 0.0 (-0.0, 0.0) | 0.00 | NA | NA | 0.0 (-0.0, 0.0) | 0.00 | NA | NA |
| S. aureus to penicillin | 2012-2018 | 2.8 (-9.6, 17.0) | 0.03 | 0.56 | 0.60 | 56.2 (34.8, 81.0) | 0.41 | 5.90 | <0.001 [c] |
| | 2018-2021 | 260.3 (114.5, 505.2) | 1.28 | 6.35 | 0.001 [c] | | | | |
| S. aureus to gentamicin | 2012-2021 | -0.1 (-0.8, 0.7) | -0.0005 | -0.17 | 0.87 | -0.1 (-0.8, 0.7) | -0.0005 | -0.17 | 0.87 |
| S. aureus to oxacillin | 2012-2021 | -1.1 (-2.4, 0.2) | -0.01 | -1.89 | 0.10 | -1.1 (-2.4, 0.2) | -0.01 | -1.89 | 0.10 |

Abbreviations: APC, annual percentage change; AAPC, average annual percentage change; CI, confidence intervals; ESBL, extended-spectrum beta-lactamase; E. coli, Escherichia coli; GBS, Group B Streptococcus; E. spp., Enterococcus spp.; S. aureus, Staphylococcus aureus; NA, not available.

[a] If the AAPC is within one segment, the t-distribution is used. Otherwise, the normal (Z) distribution is used.

[b] Proportion of ESBL-negative E. coli strains.

[c] P <0.05.
