# Supplementary material for: Trends in the antimicrobial susceptibility among Chinese neonates from 2012 to 2021: a multicenter study
Source: Antimicrob Resist Infect Control. 2024 Jul 30;13:83. doi: 10.1186/s13756-024-01440-2 (PMC11290293; doi:10.1186/s13756-024-01440-2)
Supplement: Supplementary file 4 — Additional File 4. Table of Mantel-Haenszel linear-by-linear chi-square test for MIC of antibiotics against pathogens in Chinese full-term neonates with IBIs, 2019–2021 [file 13756_2024_1440_MOESM4_ESM.pdf]

Additional file 4

Table: Mantel-Haenszel linear-by-linear chi-square test for MIC of antibiotics against pathogens in Chinese full-term neonates with IBIs, 2019-2021

	2019	2020	2021	Linear trend		Pearson-related	
E. coli [a]							
Ampicillin MIC (ug/ml), n (%)	n=17	n=13	n=12	χ^2= 0.022	P= 0.882	R= -0.023	P= 0.885
≤2	1 (5.9)	1 (7.7)	1 (8.3)				
16	7 (41.2)	3 (23.1)	5 (41.7)				
≥32	9 (52.9)	9 (69.2)	6 (50.0)				
Cefepime MIC (ug/ml), n (%)	n=26	n=20	n=17	χ^2= 0.004	P= 0.948	R= 0.008	P= 0.949
≤0.12	7 (26.9)	7 (35.0)	6 (35.3)				
0.5	7 (26.9)	0 (0.0)	5 (29.4)				
1	3 (11.5)	4 (20.0)	0 (0.0)				
2	4 (15.4)	5 (25.0)	0 (0.0)				
32	4 (15.4)	2 (10.0)	5 (29.4)				
≥64	1 (3.8)	2 (10.0)	1 (5.9)				
Gentamicin MIC (ug/ml), n (%)	n=17	n=15	n=13	χ^2= 0.335	P= 0.563	R= 0.087	P= 0.569
≤1	9 (52.9)	6 (40.0)	8 (61.5)				
4	5 (29.4)	6 (40.0)	1 (7.7)				
8	2 (11.8)	0 (0.0)	1 (7.7)				
≥16	1 (5.9)	3 (20.0)	3 (23.1)				
Cefotaxime MIC (ug/ml), n (%)	n=6	n=4	n=6	χ^2= 0.000	P= 1.000	R= 0.000	P= 1.000
0.5	0 (0.0)	0 (0.0)	1 (16.7)				
1	2 (33.3)	2 (50.0)	0 (0.0)				

Table: Mantel-Haenszel linear-by-linear chi-square test for MIC of antibiotics against pathogens in Chinese full-term neonates with IBIs, 2019-2021 (continued)

	2019	2020	2021	Linear trend		Pearson-related	
E. coli [a]							
Cefotaxime MIC (ug/ml), n (%)	n=6	n=4	n=6	χ^2= 0.000	P= 1.000	R= 0.000	P= 1.000
＞32	4 (66.7)	2 (50.0)	5 (83.3)				
Ceftriaxone MIC (ug/ml), n (%)	n=26	n=20	n=12	χ^2= 1.163	P= 0.281	R= 0.143	P= 0.285
≤0.25	7 (26.9)	7 (35.0)	2 (16.7)				
0.5	10 (38.5)	6 (30.0)	3 (25.0)				
32	4 (15.4)	1 (5.0)	3 (25.0)				
≥64	5 (19.2)	6 (30.0)	4 (33.3)				
Meropenem MIC (ug/ml), n (%)	n=7	n=12	n=13	χ^2= 1.656	P= 0.198	R= -0.231	P= 0.203
≤0.25	1 (14.3)	5 (41.7)	6 (46.2)				
0.5	6 (85.7)	7 (58.3)	7 (53.8)				
GBS [a]							
Erythromycin MIC (ug/ml), n (%)	n=10	n=6	n=6	χ^2= 1.200	P= 0.273	R= -0.239	P= 0.284
≤0.25	4 (40.0)	4 (66.7)	4 (66.7)				
≥0.5	6 (60.0)	2 (33.3)	2 (33.3)				
Clindamycin MIC (ug/ml), n (%)	n=12	n=6	n=4	χ^2= 5.815	P= 0.016 [b]	R= -0.526	P= 0.012 [b]
0.125	0 (0.0)	4 (66.7)	3 (75.0)				
0.25	2 (16.7)	0 (0.0)	0 (0.0)				
1	4 (33.3)	0 (0.0)	0 (0.0)				
≥8	6 (50.0)	2 (33.3)	1 (25.0)				

Table: Mantel-Haenszel linear-by-linear chi-square test for MIC of antibiotics against pathogens in Chinese full-term neonates with IBIs, 2019-2021 (continued)

	2019	2020	2021	Linear trend		Pearson-related	
GBS [a]							
Linezolid MIC (ug/ml), n (%)	n=8	n=6	n=8	χ^2= 1.203	P= 0.273	R= -0.239	P= 0.283
1	1 (12.5)	2 (33.3)	3 (37.5)				
2	7 (87.5)	4 (66.7)	5 (62.5)				
Vancomycin MIC (ug/ml), n (%)	n=7	n=6	n=8	χ^2= 3.245	P= 0.072	R= -0.403	P= 0.070
≤0.5	5 (71.4)	6 (100.0)	8 (100.0)				
1	2 (28.6)	0 (0.0)	0 (0.0)				
E. Spp. [a]							
Ampicillin MIC (ug/ml), n (%)	n=7	n=8	n=19	χ^2= 0.142	P= 0.706	R= -0.066	P= 0.712
≤2	2 (28.6)	3 (37.5)	10 (52.6)				
16	3 (42.9)	1 (12.5)	1 (5.3)				
≥32	2 (28.6)	4 (50.0)	8 (42.1)				
Linezolid MIC (ug/ml), n (%)	n=7	n=8	n=17	χ^2= 0.557	P= 0.456	R= -0.134	P= 0.465
≤1	7 (100.0)	1 (12.5)	16 (94.1)				
2	0 (0.0)	7 (87.5)	1 (5.9)				
Penicillin MIC (ug/ml), n (%)	n=7	n=8	n=19	χ^2= 0.003	P= 0.954	R= -0.010	P= 0.955
2	2 (28.6)	2 (25.0)	6 (31.6)				
4	0 (0.0)	1 (12.5)	2 (10.5)				
16	2 (28.6)	1 (12.5)	2 (10.5)				
32	1 (14.3)	0 (0.0)	1 (5.3)				
≥64	2 (28.6)	4 (50.0)	8 (42.1)				

Table: Mantel-Haenszel linear-by-linear chi-square test for MIC of antibiotics against pathogens in Chinese full-term neonates with IBIs, 2019-2021 (continued)

	2019	2020	2021	Linear trend		Pearson-related	
E. Spp. [a]							
High-concentration gentamicin MIC (ug/ml), n (%)	n=10	n=8	n=12	χ^2= 1.966	P= 0.161	R= -0.260	P= 0.165
≤500	8 (80.0)	6 (75.0)	12 (100.0)				
>500	2 (20.0)	2 (25.0)	0 (0.0)				
Vancomycin MIC (ug/ml), n (%)	n=7	n=8	n=18	χ^2= 1.037	P= 0.309	R= -0.180	P= 0.316
≤0.5	0 (0.0)	3 (37.5)	2 (11.1)				
1	6 (85.7)	3 (37.5)	16 (88.9)				
2	1 (14.3)	2 (25.0)	0 (0.0)				
S. aureus [a]							
Clindamycin MIC (ug/ml), n (%)	n=12	n=12	n=10	χ^2= 0.024	P= 0.878	R= 0.027	P= 0.881
≤0.25	11 (91.7)	10 (83.3)	9 (90.0)				
0.5	1 (8.3)	2 (16.7)	1 (10.0)				
Linezolid MIC (ug/ml), n (%)	n=12	n=12	n=10	NA	NA	NA	NA
2	12 (100.0)	12 (100.0)	10 (100.0)				
Vancomycin MIC (ug/ml), n (%)	n=10	n=12	n=12	χ^2= 4.435	P= 0.035 [b]	R= -0.367	P= 0.033 [b]
≤0.5	3 (30.0)	5 (41.7)	9 (75.0)				
1	7 (70.0)	7 (58.3)	3 (25.0)				
Penicillin MIC (ug/ml), n (%)	n=12	n=12	n=10	χ^2= 4.547	P= 0.033 [b]	R= -0.371	P= 0.031 [b]
≤0.12	2 (16.7)	4 (33.3)	5 (50.0)				
0.25	1 (8.3)	2 (16.7)	2 (20.0)				
0.5	8 (66.7)	6 (50.0)	3 (30.0)				
＞8	1 (8.3)	0 (0.0)	0 (0.0)				

Table: Mantel-Haenszel linear-by-linear chi-square test for MIC of antibiotics against pathogens in Chinese full-term neonates with IBIs, 2019-2021 (continued)

	2019	2020	2021	Linear trend		Pearson-related	
S. aureus [a]							
Gentamicin MIC (ug/ml), n (%)	n=12	n=12	n=10	χ^2= 0.011	P= 0.916	R= 0.018	P= 0.918
≤0.5	11 (91.7)	12 (100.0)	9 (90.0)				
4	1 (8.3)	0 (0.0)	1 (10.0)				

Abbreviations: MIC, minimum inhibitory concentration; E. coli, Escherichia coli; GBS, Group B Streptococcus; E.spp., Enterococcus spp.; S. aureus, Staphylococcus aureus; NA, not available

[a] A small portion of strains did not have recorded MIC values for certain antibiotics, which were excluded from this analysis.

[b] P <0.05.
